# Sexual double standard: A gender-based prejudice referring to sexual freedom and sexual shyness

**DOI:** 10.3389/fpsyg.2022.1006675

**Published:** 2022-10-12

**Authors:** Carmen Gómez-Berrocal, Nieves Moyano, Ana Álvarez-Muelas, Juan Carlos Sierra

**Affiliations:** ^1^Mind, Brain, and Behavior Research Center, University of Granada, Granada, Spain; ^2^Department of Evolutionary and Educational Psychology, University of Jaén, Jaén, Spain

**Keywords:** sexual double standard, gender-based prejudice, sexual gender norms, sexual freedom, sexual shyness

## Abstract

The sexual double standard (SDS) consists of judging men and women differently for the same sexual behavior. This study contributes to research on the factors that determine inconsistent adherence to the SDS. It uses a descriptive methodology to analyze the association between individual and contextual factors both with adherence to the SDS, and with four SDS adherence typologies (man-favorable SDS, woman-favorable SDS, egalitarian and ambivalent). A total of 1,206 heterosexual Spanish adults (603 men and 603 women) participated. The mean age in the male sample was 41.7 (*SD* = 14.25), in the female sample *M* = 40.84 (*SD* = 14.24). The results show that the conceptualization of SDS as a gender-based prejudice is valid to understand the bias of ingroup favoritism that SDS implies: adherence to SDS is more related to the identity of the gender role of men (vs. women). In addition, evidence is provided that the normative context and domain of sexual behavior (i.e., sexual freedom or sexual shyness) determine the form that SDS adopts to express itself. The domain of behaviors related to sexual shyness (vs. domain related to sexual freedom) better discriminates between the different four SDS adherence typologies. The importance of adopting different levels of analysis (i.e., individual, intergroup, societal) to explain and predict both SDS adherence and the prevalence of SDS adherence typologies is discussed.

## Introduction

The Sexual Double Standard (SDS) occurs when similar sexual behaviors are evaluated differently depending on whether a man or a woman performs them (Milhausen and Herold, 2002). Non-adherence to SDS implies an egalitarian gender attitude regarding sexuality. In this case, the evaluation of sexual behavior is independent of who (i.e., a man or a woman) exhibits them.

Some authors have proposed that SDS is a contextual phenomenon, as results support its existence only on certain occasions (Zaikman and Marks, 2014). For example, although the measure of SDS through self-report shows that most people report being egalitarian, different SDS adherence typologies still prevail (Álvarez-Muelas et al., 2021b). Furthermore, measures with the Implicit Association Test (IAT; Greenwald et al., 2009) show that the traditional attitude towards SDS is automatically activated (Marks, 2008; Jonason and Marks, 2009; Kreager and Staff, 2009). Therefore, it is necessary to study the factors that influence or relate to the inconsistency that characterizes the adherence to SDS. We propose that a relevant factor in this inconsistency is the domain of sexual behaviors to which the scale used to measure SDS refers.

### Adherence to SDS and domains of sexual behavior

Traditionally, SDS has been measured concerning the domain of sexual behaviors related to the exercise of sexual freedom. Adherence to SDS relative to this area reflects the degree to which the respondent recognizes and agrees that freely and openly exercising sexuality is equally desirable or beneficial for both men and women (Álvarez-Muelas et al., 2021b). Support for the so-called traditional SDS values the free and active expression of sexuality more positively in men than in women (Zaikman and Marks, 2017). In this sense, we can affirm that the prevalence of traditional SDS implies an attitude that values sexual freedom in favor of men (i.e., man-favorable SDS). However, the reverse sexual double standard, which values high sexual activity more positively in women than in men (Milhausen and Herold, 1999), implies a woman-favorable SDS.

Recently, SDS has been measured in the domain of sexual behaviors related to sexual shyness (Sierra et al., 2018; Álvarez-Muelas et al., 2021b, 2022). In this case, adherence to SDS measures to the extent to which the respondent recognizes and approves, for both men and women, the willingness and desire to manifest decorum, chastity, and continence in sexual relations (Álvarez-Muelas et al., 2021b). In the dimension related to sexual shyness, a man-favorable SDS consists in assessing sexual shyness more positively in women than in men. We consider that such a form of adherence to SDS expresses gender-based prejudice, as in democratic societies, since the mid-20th century, there is increasing agreement on the right to free expression of sexuality and premarital sex (King et al., 1977; Wells and Twenge, 2005).

This study aims to contribute to the understanding of the factors that favor the variability with which adherence to SDS manifests. To this end, we propose that SDS is a gender-based prejudice that involves a biased and negative group evaluation (e.g., women) or an individual (e.g., a particular woman) based on her group membership (i.e., the women’s collective) (Crandall et al., 2002). Notice that as it usually happens with expressions of modern prejudice (Pearson et al., 2009), SDS might adopt an ambivalent expression (Álvarez-Muelas et al., 2021b). When an attitude is ambivalent the person has both pro and con beliefs, or positive and negative emotions when evaluating sexual behavior in men and women (Albarracín et al., 2014).

### Intergroup gender relations and adherence to SDS

Conceptualizing SDS as a gender-based prejudice justifies an analysis of its adherence and prevalence within the framework of intergroup relations between men and women. From this perspective, men and women self-perceive themselves as members of a social group with which they share the same social identity (Turner and Reynolds, 2011). When the man vs. woman categorization becomes salient, both men and women interact in terms of their social identity (Turner et al., 1987), and this social identity will tend to accentuate differences between the ingroup and outgroup on dimensions of comparison that are relevant (Jetten and Spears, 2003). There is evidence that men differentially characterize the ingroup through comparison with women and that positive own group differentiation (i.e., ingroup favoritism bias) predicts sexism against women (Gómez-Berrocal et al., 2011). Therefore, the analysis of SDS adherence in an intergroup context involves testing whether the comparison with the outgroup (e.g., women) on dimensions of sexual behavior (e.g., dimensions related to sexual freedom or sexual shyness) yields favoritism toward one’s own group (e.g., men).

The existence of SDS has been questioned on the grounds that gender differences are scarce for many sexual behaviors and attitudes (Petersen and Hyde, 2010). Related to this issue, it is important to consider what beliefs the respondent is relying on to make their judgements. The categories “male” and “female” will be those that relate to responses to an SDS scale if the respondent makes judgments based on biological differences between men and women (Buss, 2006; Endendijk et al., 2020). However, the respondent may also understand that men and women are biologically the same, but different in the sex roles they perform (Herek, 1986; Klein et al., 2019). In this case, gender role-based categories (i.e., masculine, feminine, androgynous, and undifferentiated; Bem, 1974) will determine responses to a scale assessing SDS. From this approach, it is vital to explore under what conditions the SDS measure will yield differences across gender role or across man and woman categories.

On the other hand, the form that adherence to SDS takes-for example, when it assumes a man-favorable bias-may reinforce the traditional gender hierarchy that reserves decision-making power and assertiveness for men, and passivity for women (Christopher and Wojda, 2008; Rosenthal et al., 2012). Social dominance orientation (Pratto et al., 1994; Sibley et al., 2007; Asbrock et al., 2010) describes an individual characteristic that reflects a general preference for hierarchy (vs. egalitarian relationships) among groups living together in a society (Pratto et al., 1994). Previous studies have linked social dominance orientation to hostile sexism toward women (Sibley et al., 2007) and to SDS adherence that is favorable to men (Sierra et al., 2018; Gómez-Berrocal et al., 2019). Thus, we postulate that this dispositional characteristic will predict the forms of SDS adherence that favor traditional gender hierarchy.

### Contextual factors and SDS adherence

Twelve European countries already have laws to guarantee the right to sexual freedom and equality for men and women. This situation may inhibit adherence to SDS if we consider that the normative context determines the prevalence of and individual adherence to any prejudicial attitude (Crandall et al., 2002). In this regard, it has been found that the SDS adherence typologies (man-favorable SDS, woman-favorable SDS, and egalitarian) vary depending on the cultural and normative context. For example, in societies with strong democracies, the prevalence of the egalitarian typology is high (Sánchez-Fuentes et al., 2020; Álvarez-Muelas et al., 2022). Even the mere perception that one has about the consensus that supports antiprejudice norms (i.e., perceived normativity) can have an effect on personal adherence to prejudice (Sechrist and Stangor, 2001; Stangor et al., 2001). For example, perceived normativity about social support for SDS has been related to individual attitude toward SDS (Gómez-Berrocal et al., 2019).

The normative context that favors openness and sexual liberation has been assumed to be determinant in understanding the observed reduction in adherence to traditional SDS (Thompson et al., 2018). In the context of democratic societies, we assume that the norm advocating openness and the right to sexual freedom (i.e., acceptance of sexual freedom) coexists with another norm-advocating sexual equality between men and women (i.e., the norm of non-adherence to SDS). Attitudes toward sexual freedom in general (e.g., acceptance of sexual freedom) will make the modern vs. old-fashioned categorisation salient, and attitudes toward sexual equality between men and women will salience gender categorization (i.e., man vs. woman) and probably competition intergroup (Ellemers and Haslam, 2011; Turner and Reynolds, 2011). From this approach, we expect that there will be no differences between men and women in responses to a scale that generally measures acceptance of sexual freedom. However, there will probably be differences between men and women in responses to a scale measuring SDS, as such a scale may capture motivation towards ingroup distinctiveness through ingroup favoritism (Tajfel and Turner, 1986; Dovidio and Gaertner, 2010).

On the other hand, the domain of sexual behavior (i.e., sexual freedom and sexual shyness) concerning the measures of SDS is a determinant factor of the variability shown by SDS. In order to describe the prevalence in Spain of different forms of SDS adherence in relation to both sexual freedom and sexual shyness, Álvarez-Muelas et al. (2021b) found that the percentage of people with an egalitarian standard was higher in the domain of behaviors related to sexual freedom compared to in the domain of behaviors related to sexual shyness. Adherence to prejudice is more subtle and more politically correct when it implies an ingroup favoritism bias on positive dimensions or domains (i.e., that society does not censor) but not on negative dimensions or domains (i.e., that society censors; Dovidio et al., 2016). It seems logical to assume that in democratic societies, gender equality will be frowned upon, even censored, when it refers to behaviors related to sexual freedom. However, there is no clear normative context in the sexual shyness area. It is therefore crucial to know whether the SDS expression concerning the area of sexual shyness represents a more subtle form of prejudice in the context of sexual openness that characterizes democratic societies.

Finally, the need to maintain gender differences is likely to change over the individual’s evolutionary development. The evidence shows that people in different age groups express a favourable attitude towards the sexual double standard: adolescents (Monge et al., 2013), young adults (Sakaluk and Milhausen, 2012), and over 50 years old (Sierra et al., 2010). In non-English speaking samples, people older than 50 years old have been found to report higher mean scores in adherence to SDS in favor of men (Sierra et al., 2018). However, men aged 26–55 years and women older than 56 (Álvarez-Muelas et al., 2021b) entail the highest percentage of people supporting man-favorable SDS.

Against this background, the main objective of this article is to describe the processes associated with the expression of adherence to SDS. Two hypotheses and three research questions are proposed.

*H1*: SDS is a gender-based prejudice that reflects motivations to achieve a positive gender identity for the ingroup (Tajfel and Turner, 1986). Adherence to SDS is expected to be a result of the interaction between gender (i.e., men vs. women) and gender role (i.e., masculine, feminine, androgynous, and undifferentiated).

*RQ1*: Are acceptance of sexual freedom and acceptance of sexual shyness associated with the respondent's gender and gender role?

*RQ2*: How is acceptance of sexual freedom and sexual shyness associated with the forms that adherence to SDS takes (man-favorable SDS, woman-favorable SDS, egalitarian, and ambivalent)?

*H2*: It is expected to find a significant correlation between adherence to SDS and the perceived normativity on sexual gender roles. Individual adherence to SDS will be lower if the social environment is perceived not to support SDS.

*RQ3*: To what extent do individual (i.e., sex, age, gender role, and social dominance orientation) and contextual (i.e., perceived normativity of sexual gender norms) factors explain the types of SDS adherence (i.e., man-favorable SDS, woman-favorable SDS, egalitarian, and ambivalent) in the domains of sexual freedom and sexual shyness?

## Materials and methods

### Participants

The sample was composed of 1,206 individuals who fulfilled the following criteria: (a) aged ≥18 years; (b) Spanish nationality; and (c) heterosexual. Participants were recruited from the general Spanish population. A quota convenience sampling method was used to obtain the required number of men (*n* = 603) and women (*n* = 603), whose mean age was 41.7 (*SD* = 14.25) for men and 40.84 (*SD* = 14.24) for women. Most of the participants had a university degree and were in a relationship. To fulfill the goals of the present study, the sociodemographic information of the participants is shown based on the four SDS adherence typologies (man-favorable SDS, woman-favorable SDS, egalitarian, and ambivalent),on both sexual freedom and sexual shyness in Table 1, and on gender roles (masculine, feminine androgynous, and undifferentiated) in Table 2. In terms of gender roles, men identified more with a masculine role compared to women, who defined themselves as more feminine. Finally, more men than women characterized themselves as having an undifferentiated role.

**Table 1:** Sociodemographic characteristics of the sample according to SDS adherence typologies for sexual freedom and sexual shyness.

	Typologies of SDS adherence for sexual freedom				Typologies of SDS adherence for sexual shyness			
	Man-favorable (*n* = 369)	Woman-favorable (*n* = 339)	Egalitarian (*n* = 420)	Ambivalent (*n* = 78)	Man-favorable (*n* = 388)	Woman-favorable (*n* = 287)	Egalitarian (*n* = 437)	Ambivalent (*n* = 94)
*Gender*								
Men (%)	64.2	38.6	47.1	47.4	64.9	41.5	43.7	43.6
Women (%)	35.8	61.4	52.9	52.6	35.1	58.5	56.3	56.4
*Education*								
No formal studies (%)	2.4	1.8	1.2	1.3	1.3	1.7	1.6	4.3
Primary education (%)	12.5	9.1	6.7	7.7	13.7	9.4	5.7	6.4
High school (%)	24.9	24.2	20	28.2	26.3	25.1	18.5	26.6
University degree (%)	59.1	64.6	71.7	62.8	58.5	62.7	73.7	61.7
*In a relationship*								
Yes (%)	78.3	76.1	76.4	74.4	77.8	79.1	74.122.7	77.7
No (%)	18.7	21.2	20.2	23.1	20.1	16.7		20.2

**Table 2:** Sociodemographic characteristics of the sample according to gender roles.

	Gender roles			
	Masculine (*n* = 369)	Feminine (*n* = 339)	Androgynous (*n* = 420)	Undifferentiated (*n* = 78)
*Gender*				
Men (%)	55.7	39.1	45.3	56.5
Women (%)	44.3	60.9	54.7	43.5
*Education*				
No formal studies (%)	0.4	3	0.9	2
Primary Education (%)	9.4	11.2	8.5	8.1
High school (%)	19.7	25.7	24.1	23.1
University degree (%)	69.3	59.9	66	66.4
*In a relationship*				
Yes (%)	74.2	79.3	76.9	76.5
No (%)	23	18.4	21.7	19.3

### Instruments

Socio-demographic questionnaire. It includes questions about gender (i.e., man vs. woman), age, nationality, sexual orientation, education, and partner relationship among others.

The Spanish version of the Sexual Double Standard Scale (Muehlenhard and Quackenbush, 2011; Sierra et al., 2018). It consists of 16 items with a 4-point Likert-type response scale from 0 *(strongly disagree)* to 3 *(strongly agree),* which are structured into two factors of 8 items each: Acceptance of sexual freedom and Acceptance of sexual shyness. The eight items for each factor are written in parallel: four refer to sexual behaviors attributed to men and four to sexual behaviors attributed to women. From the response to the items of each factor, an index of adherence to the SDS can be obtained. The Index of Double Standard for Sexual Freedom is obtained from the Acceptance of sexual freedom factor and is the result of subtracting from the score in the four items referring to men the score in the four items referring to women. Similarly, the Index of Double Standard for Sexual Shyness is obtained from the factor Acceptance of sexual shyness and is the result of subtracting from the score in the four items referring to women the score in the four items referring to men. The Index of Double Standard for Sexual Freedom and the Index of Double Standard for Sexual Shyness are bipolar measures, their scores range from −12 to +12, and a neutral score is equal to 0. In both indices, negative scores represent adherence to SDS in favor of women, and positive scores represent adherence to SDS in favor of men. Specifically, for the Index of Double Standard for Sexual Freedom, negative scores (−1 to −12) indicate adherence to a sexual double standard more in favor of sexual freedom in women than in men; positive scores (+1 to +12) indicate adherence to an SDS more in favor of sexual freedom in men than in women. For the Index of Double Standard for Sexual Shyness, negative scores (−1 to −12) indicate adherence to a sexual double standard more in favor of sexual shyness in men than in women; positive scores (+1 to +12) indicate adherence to an SDS more in favor of sexual shyness in women than in men. From the Index of Double Standard for Sexual Freedom and Index of Double Standard for Sexual Shyness, four types of adherence to SDS are obtained: man-favorable SDS, woman-favorable SDS, egalitarian and ambivalent referred to sexual freedom and sexual shyness, respectively. The man-favorable SDS typology includes those people with positive scores (between +1 and + 12) on both indices. In the Index of Double Standard for Sexual Freedom, the man-favorable SDS typology represents supporting and defending greater sexual freedom for men than for women. The Index of Double Standard for Sexual Shyness represents supporting less sexual shyness for men than for women. The woman-favorable SDS typology is obtained from the scores that take a negative value on both indices (between −1 and − 12). In the Index of Double Standard for Sexual Freedom, the woman-favorable SDS typology represents defending greater sexual freedom for women than for men, while the Index of Double Standard for Sexual Shyness defends less sexual shyness for women than for men. The egalitarian typology includes those people whose score equals zero in either the Index of Double Standard for Sexual Freedom or Index of Double Standard for Sexual Shyness and, in turn, who obtain a zero result in the subtractions between the pairs of parallel items that make up either of these two indices. This typology includes those people who defend the same criterion for men and women alike when evaluating behaviors referring to both sexual freedom and sexual shyness. Finally, the ambivalent typology groups those people with a zero score in the Index of Double Standard for Sexual Freedom or Index of Double Standard for Sexual Shyness, and who obtain non-zero results in some items that make up either of these two indices. This typology includes those people who obtain inconsistent scores when evaluating sexual behaviors referring to sexual freedom or sexual shyness. The scale showed suitable internal consistency (ordinal alpha 0.84 for the Acceptance of the sexual freedom factor and 0.87 for the Acceptance of the sexual shyness factor), and its test–retest reliability coefficients were above 0.70 at 4 and 8 weeks (Sierra et al., 2018). It also proved to be invariant by gender and age (Álvarez-Muelas et al., 2019). In this study, the ordinal alpha values obtained were 0.79 and 0.82 for the Acceptance of sexual freedom, and 0.71 and 0.70 for the Acceptance of sexual shyness in men and women, respectively.

The Spanish version of Bem Sex Role Inventory (Bem, 1974; Fernández et al., 2007), the short version adapted by Gómez-Berrocal et al. (2022) is used. By means of eight items it assesses the gender role as a self-description according to a series of personality traits of the gender stereotype: four items represent the masculine dimension (e.g., behaves like a leader, has leadership abilities, dominant, and strong personality) and four items represent the feminine dimension (e.g., sensitive to needs of others, compassionate, gentle, and affectionate). The response scale is Likert-type from 1 (*never*) to 7 (*always*). The scores are used to obtain a Masculinity and a Femininity index, from which the person is classified as masculine, feminine, androgynous and undifferentiated according to the participants self-description in terms of the characteristics of both dimensions. The scale showed adequate internal consistency, with Cronbach’s alpha coefficients of 0.84 for Masculinity and 0.75 for Femininity (Gómez-Berrocal et al., 2022). In this study, alpha coefficients were equal to 0.73 and 0.73 for Femininity in men and women, respectively, and 0.79 for Masculinity in both genders.

The Spanish version of Social Dominance Orientation Scale (Pratto et al., 1994; Silván-Ferrero and Bustillos, 2007). It consists of 16 items that are answered on a 7-point Likert scale from 1 (*completely disagree*) to 7 (*completely agree*), and two factors: Opposition to equality (*α* = 0.84) and Group-based dominance (*α* = 0.77) (Silván-Ferrero and Bustillos, 2007). In this study, ordinal alpha coefficients were 0.69 in men and 0.68 in women, and 0.76 in men and 0.74 in women for the two factors, respectively.

The Spanish hetero-referred version of Sexual Double Standard Scale (Muehlenhard and Quackenbush, 2011) by Gómez-Berrocal et al. (2019). It measures perceived normativity, that is, the perceived degree to which society accepts certain gender norms about sexual behaviors. The scale is composed of 18 items that are answered on a 4-point Likert-type scale, from 0 (*strongly disagree*) to 3 (*strongly agree*), and three factors: Social acceptance of man sexual shyness, Social acceptance of woman sexual freedom, and Social acceptance of sexual double standards. For each factor, internal consistency obtained ordinal alpha values of 0.73, 0.70 and 0.90, respectively. In our sample, the ranges of values were 0.67 and 0.69 in men and women for Social acceptance of man sexual shyness, 0.66 and 0.63 in men and women for Social acceptance of woman sexual freedom, and 0.75 and 0.66 for Social acceptance of sexual double standard.

### Procedure

A nonrandom sampling procedure was applied to the general Spanish population to recruit the participants. Questionnaires were administered in paper and pencil format (84.2%) by two evaluators in different universities, social centers, and associations, and *via* an online format (15.8%). Regarding the paper and pencil format, participants completed the scales alone and in private, and returned them *via* a sealed envelope. Regarding the online format, the URL of the questionnaires was distributed through social networks and the news service of the University of Granada. Information on general sexual behaviors did not differ by questionnaire modality (Sierra et al., 2018; Álvarez-Muelas et al., 2021a). The subjects were informed of the purpose and procedure of the study. All participants were assured of the anonymity and confidentiality of the data. The time to complete the questionnaires was estimated at 30 min. This research was approved by the Ethics Committee of Human Research of the University of Granada, Spain.

### Data analysis

To examine whether there were significant differences by gender and by gender roles, both indices of SDS adherence (i.e., Index of Double Standard for Sexual Freedom and Index of Double Standard for Sexual Shyness) and acceptance of sexual freedom and acceptance of sexual shyness we conducted MANOVA. Pairwise comparisons between the different gender roles were performed.

To examine differences in acceptance of sexual freedom and acceptance of sexual shyness by typologies of SDS adherence (man-favorable SDS, woman-favorable SDS, egalitarian, and ambivalent) we conducted univariate ANOVAs. To find out to what extent SDS adherence is related to perceived normativity about sexual gender roles, correlations were conducted between each factor of the hetero-referred scale of the SDS and the indices of SDS adherence. Since both indices are bipolar measures, correlations were conducted separately for negative scores (from −1 to −12) and for positive scores (from 0 to +12). For both indices, negative scores represent adherence with a woman-favorable SDS and positive scores represent adherence with a man-favorable SDS.

Finally, logistic regression analyses were conducted to determine the explanatory power of individual variables (i.e., sex, age, gender roles, opposition to equality and group-based dominance) and normative variables (i.e., social acceptance of man sexual shyness, social acceptance of woman sexual freedom, and social acceptance of sexual double standard) on the four typologies of SDS adherence (i.e., man-favorable SDS, woman-favorable SDS, egalitarian, and ambivalent) in two domains of sexual behavior (sexual freedom and sexual shyness).

## Results

### Adherence to SDS with respect to sexual freedom and sexual shyness: Differences across gender and gender role (H1)

#### Differences between men and women

We found significant differences between men and women for both the Index of Double Standard for Sexual Freedom (*p* < 0.001) and Index of Double Standard for Sexual Shyness (*p* < 0.01) scores. Regarding the first index, both men and women had positive scores, although they were higher in men; men reported ingroup favoritism and women outgroup favoritism. For the second index, men had positive scores and women had negative scores; therefore, both displayed ingroup favoritism (Table 3).

**Table 3:** Differences in Index of Double Standard for Sexual Freedom, Index of Double Standard for Sexual Shyness, Acceptance of sexual freedom and Acceptance of sexual shyness across gender and gender roles.

	Global		Feminine		Masculine		Androgynous		Undifferentiated			
	Men	Women	Men	Women	Men	Women	Men	Women	Men	Women	Gender roles	Gender
	*M (SD)*	*M (SD)*	*M (SD)*	*M (SD)*	*M (SD)*	*M (SD)*	*M (SD)*	*M (SD)*	*M (SD)*	*M (SD)*	*F*_(3, 599)_	*F*_(1, 1,204)_
IDS-SF	2.27 (2.60)_a_	1.42 (2.44)_b_	1.80 (2.57)_a, b, c_	1.43 (2.39)	2.93 (2.59)_a_	1.66 (2.39)	2.47 (2.91)_b_	1.52 (2.72)	2.07 (2.40)_c_	1.14 (2.29)	1.09	34.13^***^
IDS-SS	0.62 (1.89)_a_	−0.06 (1.42)_b_	0.28 (1.52)_a, b_	0.02 (1.37)	1.14 (2.46)_a_	−0.27 (1.41)	0.56 (1.99)_b_	0.13 (1.46)	0.54 (1.59)_a, b_	−0.22 (1.45)	2.55	50.51^***^
A-SF	11.42 (4.65)	10.96 (4.95)	10.71 (5.09)_b_	10.34 (4.92)_b_	12.56 (4.12)_a_	11.59 (5.08)_a_	11.08 (5.21)_a, b_	11.24 (5.44)_a, b_	11.34 (4.27)_a, b_	11.20 (4.40)_b_	2.00	2.71
A-SS	6.89 (4.73)_a_	5.99 (4.74)_b_	6.77 (5.00)	5.98 (4.83)	7.07 (4.60)	5.53 (4.64)	7.21 (5.33)	6.12 (5.31)	6.73 (4.34)	6.22 (4.16)	0.47	11.10^***^

IDS-SF: Index of Double Standard for Sexual Freedom; IDS-SS: Index of Double Standard for Sexual Shyness; A-SF: acceptance of sexual freedom; A-SS: acceptance of sexual shyness. Different subscript letters indicate the groups that significantly differ within the male and female sample separately.

*** *p* < 0.001.

#### Differences by gender role

We only found significant differences in the men sample. All four gender roles had positive scores on both the Index of Double Standard for Sexual Freedom and Index of Double Standard for Sexual Shyness, which indicates ingroup favoritism. The significantly higher scores correspond to the masculine gender role category in the men’s sample (vs. feminine, androgynous and undifferentiated). In the women’s sample, no significant differences among gender role categories were found (Table 3).

### Acceptance of sexual freedom and sexual shyness: Differences across gender and gender role (RQ1)

#### Differences between men and women

We do not find any differences between men and women regarding the acceptance of sexual freedom. As for the acceptance of sexual shyness, we see significant differences between men and women (*p* < 0.001): men were more in favor of sexual shyness than women (Table 3).

#### Differences by gender role

Regarding acceptance of sexual freedom, differences among gender roles were found only in the sample of men. The masculine gender role had higher scores in the men’s sample (vs. feminine and undifferentiated role) for sexual freedom. Regarding acceptance of sexual shyness, we found no differences among roles neither in the sample of men nor in that of the women (Table 3).

### Acceptance of sexual freedom and sexual shyness: Differences across SDS adherence typologies (RQ2)

#### Differences across SDS adherence typologies that relate to the domain of sexual freedom

Regarding acceptance of sexual freedom, participants with an egalitarian typology in the domain of sexual freedom scored significantly higher (*M* = 11.72) than those with a man-favorable SDS typology (*M* = 10.74; *p* = 0.026). Regarding acceptance of sexual shyness, participants with an egalitarian typology scored significantly lower (*M* = 5.56) than those with the man-favorable SDS (*M* = 7.84) and ambivalent typologies (*M* = 7.31; *p* < 0.001). In addition, participants with a man-favorable SDS typology viewed sexual shyness more favorably (*M* = 7.84) than those with a woman-favorable SDS typology (*M* = 5.81; *p* ≤ 0.001; Table 4).

**Table 4:** Differences in sexual freedom and sexual shyness acceptance across SDS adherence typologies.

			Sexual freedom domain						Sexual shyness domain			
			Man-favorable (*n* = 369)	Woman-favorable (*n* = 339)	Egalitarian (*n* = 420)	Ambivalent (*n* = 78)			Man-favorable (*n* = 388)	Woman-favorable (*n* = 287)	Egalitarian (*n* = 437)	Ambivalent (*n* = 94)
	*F*	*p*	*M* (*SD*)	*M* (*SD*)	*M (SD)*	*M (SD)*	*F*	*p*	*M* (*SD*)	*M* (*SD*)	*M (SD)*	*M (SD)*
A-SF	3.09	0.026	10.74 (4.37)_a_	11.17 (4.42)	11.72 (5.51)_a_	10.67 (4.09)	8.34	<0.001	10.34 (4.66)_a_	11.08 (4.60)	12 (5.00)_a_	11.37 (4.25)
A-SS	19.09	<0.001	7.84 (4.96)_a_	5.81 (4.39)_a_	5.56 (4.51)_a. b_	7.31 (4.95)_b_	19.09	<0.001	7.66 (4.69)_a_	7.22 (4.29)_b_	4.75 (4.80)_a.b.c_	6.91 (3.82)_c_

A-SF: Acceptance of sexual freedom; A-SS: Acceptance of sexual shyness. Different subscript letters indicate the groups that significantly differ.

#### Differences across SDS adherence typologies that relate to the domain of sexual shyness

Regarding acceptance of sexual freedom, participants with an egalitarian typology scored significantly higher (*M* = 12) than those with a man-favorable SDS typology (*M* = 10.34; *p* ≤ 0.001). Regarding acceptance of sexual shyness, participants with an egalitarian typology scored significantly lower (*M* = 4.75) than those with the man-favorable SDS, woman-favorable SDS, and ambivalent typologies (*p* ≤ 0.001). Likewise, those who scored highest for acceptance of sexual shyness had a man-favorable SDS (*M* = 7.66; Table 4).

### SDS adherence and perceived normativity regarding sexual gender roles (H2)

Regarding the relationship between perceived normativity and adherence to SDS (Table 5), perceived normativity regarding women’s sexual freedom was negatively related to scores on the Index of Double Standard for Sexual Freedom (*r* = −0.063; *p* = 0.033) and the Index of Double Standard for Sexual Shyness (*r* = −0.071; *p* = 0.016). Perceived normativity about SDS was positively related to scores on the Index of Double Standard for Sexual Freedom (*r* = 0.108; *p* = 0.001) and the Index of Double Standard for Sexual Shyness (*r* = 0.116; *p* < 0.001). No significant correlation was found between perceived normativity and scores on the Index of Double Standard for Sexual Freedom and Index of Double Standard for Sexual Shyness.

**Table 5:** Correlations between the Index of Double Standard for Sexual Freedom and Index of Double Standard for Sexual Shyness with index of perceived normativity of sexual gender norms.

	AMSS	AFSF	ASDS
IDS-SF (0 a + 12)	0.008	−0.063^*^	0.108^**^
IDS-SF (−1 a − 12)	0.075	−0.066	0.044
IDS-SS (0 a + 12)	−0.025	−0.071^*^	0.116^***^
IDS-SS (−1 a − 12)	−0.02	−0.058	0.003

IDS-SF: Index of Double Standard for Sexual Freedom; IDS-SS: Index of Double Standard for Sexual Shyness; AMSS: normativity of man sexual shyness; AFSF: normativity of woman sexual freedom; ASDS: normativity of sexual double standard. ^*^p < 0.05; ^**^p < 0.01; ^***^p < 0.001.

### Regression models by SDS adherence typologies (RQ3)

#### Sexual freedom domain

Table 6 provides the results of the regression models for sexual freedom. In the man-favorable SDS typology, variables with explanatory power included age (*B* = 0.02, *SE* = 0.00, *OR* = 1.01, *p* < 0.001) and group-based dominance (*B* = 0.04, *SE* = 0.00, *OR* = 1.04, *p* < 0.001), as well as one normative variable, i.e., social acceptance of SDS (*B* = 0.02, *SE* = 0.01, *OR* = 1.02, *p* = 0.041).

**Table 6:** Hierarchical logistic regressions of predictors of the SDS adherence typologies referring to the sexual freedom domain.

	Predictor	*B*	*SE (B)*	*ΟR*	*p*	95% CI for OR	*χ* ^2^	Nagelkerke *R*^2^
Man-favorable	Gender	−0.75	0.13	0.47	0.000	0.36–0.60	77.57^***^	0.088
	Age	0.02	0.00	1.01	0.000	1.01–1.03		
	Group-based dominance	0.04	0.00	1.04	0.000	1.02–1.05		
	Normativity of acceptance of sexual double standard	0.02	0.01	1.02	0.041	1.00–1.04		
Woman-favorable	Gender	0.62	0.13	1.87	0.001	0.36–0.89	42.91^***^	0.050
	Age	−0.01	0.00	0.98	0.005	0.97–0.99		
	Normativity of acceptance of sexual double standard	−0.03	0.01	0.96	0.002	−0.05–−0.01		
Egalitarian	Group-based dominance	−0.03	0.00	0.97	0.000	0.96–1.40	20.93^***^	0.024
Ambivalent								

Only significant predictors are included in the table. OR: odds ratio; CI: confidence interval.

*** *p* < 0.001.

The variables with explanatory power for the woman-favorable SDS typology were two individual characteristics, specifically, gender (*B* = 0.62, *SE* = 0.13, *OR* = 1.87, *p* < 0.001) and age (*B* = −0.01, *SE* = 0.00, *OR* = 0.98, *p* = 0.005), and the social acceptance of SDS (*B* = −0.03, *SE* = 0.01, *OR* = 0.96, *p* = 0.002).

The egalitarian typology was only predicted by negative scores for the individual characteristic of group-based dominance (*B* = −0.03, *SE* = 0.00, *OR* = 0.97, *p* = 0.0005).

Finally, for the ambivalent typology, no variable had significant explanatory power.

#### Sexual shyness domain

Table 7 presents the results of regression models of sexual shyness. Regarding the man-favorable SDS typology, the personal variables with explanatory power were gender (*B* = −0.84, *SE* = 0.13, *OR* = 1.43, *p* = 0.001; i.e., more men than women supported man-favorable SDS), age (*B* = 0.01, *SE* = 0.00, *OR* = 1.01, *p* = 0.024) and group-based dominance (*B* = 0.03, *SE* = 0.00, *OR* = 1.03, *p* < 0.001).

**Table 7:** Hierarchical logistic regressions of predictors of the SDS adherence typologies referring to the sexual shyness domain.

	Predictor	*B*	*SE (B)*	*ΟR*	*p*	95% CI for OR	*χ* ^2^	Nagelkerke *R*^2^
Man-favorable	Gender	−0.84	0.13	0.43	0.000	0.33–0.55	70.96^***^	0.080
	Age	0.01	0.00	1.01	0.024	1.00–1.02		
	Group-based dominance	0.03	0.00	1.03	0.000	1.01–1.04		
Woman-favorable	Gender	0.49	0.14	1.63	0.000	1.24–2.14	19.01^**^	0.023
	Normativity of acceptance of man sexual shyness	0.06	0.03	1.06	0.045	1.00–1.12		
Egalitarian	Gender	−0.29	0.12	1.34	0.017	1.05–1.71	45.23^***^	0.050
	Group-based dominance	−0.03	0.00	0.96	0.000	0.95–0.98		
Ambivalent								

Only significant predictors are included in the table. OR: odds ratio; CI: confidence interval.

** *p* < 0.01;

*** *p* < 0.001.

Regarding the woman-favorable SDS typology, the factors with explanatory power were gender (*B* = 0.49, *SE* = 0.14, *OR* = 1.63, *p* = 0.000) and social acceptance of man sexual shyness (*B* = 0.06, *SE* = 0.03, *OR* = 1.06, *p* = 0.045).

Regarding the egalitarian typology, the personal variables with explanatory power were gender (*B* = −0.29, *SE* = 0.12, *OR* = 1.34, *p* = 0.017) and group-based dominance (*B* = −0.03, *SE* = 0.00, *OR* = 0.96, *p* = 0.000).

## Discussion

The Sexual Double Standard (DSP) consists of evaluating similar sexual behaviors differently depending on whether they are carried out by a man or a woman (Milhausen and Herold, 2002). Accumulating research indicates that studying the prevalence of SDS is not a trivial issue. Among the factors that hinder the evaluation of its existence, the relationship between adherence to SDS and the cultural and normative context has been pointed out (Zaikman and Marks, 2014; Habarth et al., 2019), likewise, the results on the prevalence of SDS depend on the theoretical framework and the methodology adopted by the researcher (Endendijk et al., 2020). However, equality between men and women in the field of sexuality has not yet been fully achieved. This research analyzed the association between SDS adherence and various SDS adherence typologies (man-favorable SDS, woman-favorable SDS, egalitarian, and ambivalent) with factors of individual (i.e., gender, gender role, social dominance orientation, and age) and contextual nature (i.e., perceived normativity about gendered sexual norms and domains of sexual behavior: sexual freedom and shyness). To this end, we postulate that SDS is a gender-based prejudice in the domain of sexual behaviors related to sexual freedom and sexual shyness.

Based on the assumption that men’s and women’s evaluation of sexual behavior (e.g., the measure of SDS) may reflect motivations to achieve positive differentiation from the ingroup compared to the outgroup (Tajfel and Turner, 1986), we analyzed differences across gender (i.e., man vs. woman) and gender role (i.e., masculine, feminine, androgynous, and undifferentiated) on an SDS measure related to the domain of sexual freedom and sexual shyness (H1). The results showed significant differences between men and women, but both men and women rated sexual freedom more positively in men than in women. That is, on the Index of Double Standard for Sexual Freedom measure men expressed ingroup favoritism and women expressed outgroup favoritism. This result is consistent with the postulates of social identity theory according to which ingroup favoritism bias is a strategy for ingroup differentiation more prevalent in high-status groups (Tajfel and Turner, 1986; Rubin and Hewstone, 2004). In the SDS measure of sexual shyness (Index of Double Standard for Sexual Shyness), we found differences between men and women too, but in this case, both showed ingroup favoritism. In women, this ingroup favoritism may be because they consider the hierarchy implied by SDS in behaviors related to sexual shyness to be illegitimate and unstable.

The male sample was the only one exhibiting differences across gender roles on the two measures of SDS (i.e., Index of Double Standard for Sexual Freedom and Index of Double Standard for Sexual Shyness). Men with any gender role type (masculine, feminine, androgynous, and undifferentiated) support SDS with an ingroup favoritism bias on both sexual freedom and sexual shyness behaviors. In addition, masculine-role in men is the most strongly expressing ingroup favoritism. The result is consistent with previous studies showing that men’s identification with the traditional masculine role was related to the tendency to maintain the position of privilege in the social hierarchy (Herek, 1986; Vandello and Bosson, 2013). The joint result obtained for men and women seems to coincide with that of other studies showing that gender-based prejudice is more strongly related to men’s gender self-esteem than to women’s (Falomir-Pichastor and Mugny, 2009).

We posited that the norm of acceptance of sexual freedom is probably insufficient to explain the reduction in SDS adherence (Thompson et al., 2018). We assume that the acceptance of sexual freedom, unlike SDS adherence, need not activate social gender categorization and, by the same token, neither does ingroup differentiation motivation (Tajfel and Turner, 1986; Ellemers and Haslam, 2011). To test this assumption we explored differences by gender and gender role in responses to a measure of acceptance of sexual freedom and to another of sexual shyness (RQ1). Our results showed no differences between men and women on acceptance of sexual freedom. This outcome indicates that attitude toward sexual freedom, unlike adherence to SDS in the domain of sexual freedom, is not biased by a motivation for ingroup distinctiveness. Men expressed significantly greater agreement with acceptance of sexual shyness than women. Differences by gender role were only found in men for acceptance of sexual freedom, where those with masculine roles reported more agreement with sexual freedom. Therefore, in light of the results derived from RQ1, we underscore the importance of analyzing SDS inconsistency in its intergroup and normative context. Although this study is descriptive and exploratory, it seems to show that the interpretation of ideologies enacting sexual openness depends on the intergroup context, in line with previous studies. Hence, the consequences that ideology has on intergroup attitudes, e.g., adherence to SDS, may adopt a diverse pattern (Guimond et al., 2013; Falomir-Pichastor et al., 2017).

While democratic societies may support the free exercise of sexuality, there are no similar norms regarding sexual shyness. In this regard we explored the pattern of response to acceptance of sexual freedom and acceptance of sexual shyness across types of SDS adherence (i.e., man-favorable SDS, woman- favorable SDS, egalitarian, and ambivalent; RQ2). Acceptance of sexual freedom discriminated less between types of SDS adherence than acceptance of sexual shyness. In fact, differences were found between the four SDS adherence typologies of the two domains of sexual behavior (sexual freedom and sexual shyness) in the responses to acceptance of sexual shyness. These results confirm that the dimension of sexual behaviors related to demureness discriminates better between the different forms that SDS can take, perhaps because there is no clear normative context regarding this domain of sexual behaviors (Dovidio and Gaertner, 2004; Dovidio et al., 2016).

We assume that the normative context determines individual adherence to any prejudiced attitude (Crandall et al., 2002). Thus, we described the relationship between adherence to SDS related to sexual freedom and sexual shyness and perceived normativity about sexual gender norms (H2). The results show that when the normative social context sanctions SDS (e.g., upholding women’s sexual freedom), the adherence to SDS that preserves the heteronormative pattern is inhibited (e.g., supporting more sexual freedom or less sexual shyness for men than for women). Likewise, when the normative social context endorses SDS, adherence to SDS that preserves the heteronormative pattern is favored. However, sexual gender norms are not related, at least in this sample, to adherence to what some authors have called “reverse SDS” (see Milhausen and Herold, 2002), that is, advocating more freedom or less sexual shyness for women than for men. Altogether, the results indicate that sexual gender norms appear to be related only to adherence to SDS that favors men.

Finally, we explored the explanatory power of some factors of an individual nature (i.e., gender, gender role, social dominance orientation, and age) and others of a contextual nature (i.e., normativity about sexual gender norms) on the types of SDS adherence (man-favorable SDS, woman-favorable SDS, egalitarian, and ambivalent) related to sexual freedom and sexual shyness (RQ3).

The man-favorable SDS typology, in the domain of behaviors related to sexual freedom, was explained by both individual and normative factors. Adherence to this typology is greater (1) in men than in women, (2) in the elderly than in the youth, (3) in those scoring higher in disposition to group-based dominance, and (4) as the perceived normativity of SDS increases. In the sexual shyness domain, the individual explanatory factors for the man-favorable SDS typology were the same, but no normative factor predicted man-favorable SDS.

The woman-favorable SDS typology, in the domain of behaviors related to sexual freedom, was explained by individual and normative factors. It is advocated by more women than men, its adherence is greater as the age of the participant decreases and the perceived normativity of SDS decreases. Regarding the behaviors related to sexual shyness, woman-favorable SDS is advocated by more women than men and its adherence is higher as the perceived normativity on the acceptance of sexual shyness in men increases.

The egalitarian typology, both in the domain of sexual freedom and sexual shyness behaviors, was negatively explained by an individual factor: the disposition to group-based dominance. In addition, gender predicted an egalitarian attitude toward sexual shyness, that is to say, more women than men support this typology.

Overall, the results derived from RQ3 allow us to draw several conclusions. First, it is important to study the forms of SDS adherence from different levels of analysis (Murray, 2000), as factors of a diverse nature predict different adherence forms to SDS. In addition, the sexual behaviors scope on which SDS evaluates relates to the predictors of the SDS typologies. Second, social norms determine the expression of SDS mainly in the domain of behaviors related to sexual freedom, suggesting that new forms of SDS adherence may be emerging in the sexual shyness domain. Furthermore, the results suggest the importance of analyzing sexual gender norms as an antecedent factor of heteronormative scripts that support gender-based prejudice (Habarth et al., 2019). Third, gender was found to be a predictor of all forms of SDS adherence, except for the egalitarian typology in the sexual freedom domain. The predictive ability of gender can be interpreted as showing that SDS is a gender-based prejudice whose support implies some motivation to favour the ingroup. Fourth, as age increases, man-favorable SDS prevails more, both in behaviors related to sexual freedom and shyness, and a lower prevalence of SDS in favor of women. The age variable implies more than a demographic variable indicative of the evolutionary stage of the person, as it also reflects cultural gender socialization (Donnelly and Twenge, 2017). The participants in this study, all Spaniards, had a mean age of around 40 years, that is, born in the late 1970s. However, the standard deviation of the sample (*SD* = 14) indicates that they were born between 1963 and 2000, a period during which Spain experienced a profound cultural transformation in values and norms regarding gender relations.

## Limitations and future research directions

Some of the main strengths of this study were the characteristics of the sample, for instance, it was recruited from the general Spanish population and represented both genders, different age groups and educational backgrounds. Second, we used reliable and valid measures that were adapted specifically for the population from which our sample was drawn.

One limitation of this study is the descriptive methodology used. The total size of the sample guarantees the statistical validity of the results. However, future experimental investigations should corroborate the causal relationship between the factors that we have analyzed and the forms that adherence to SDS adopts.

Our findings leave open some research questions. For example, why does adherence to SDS sometimes discriminate between men and women, and sometimes discriminate based on gender role? We propose to continue studying SDS from a gender identity perspective. Moreover, future research should study SDS in non-heterosexual samples. It should also be investigated whether the dimension of behaviors related to sexual shyness is less reactive in capturing new forms of adherence to SDS.

Future research should continue to explore the role of gender norms, relating to both sexual freedom and sexual shyness, in SDS adherence. Likewise, having participants from different generational cohorts will contribute to knowing the weight they have on the disposition to social domination gender-based, age and differential socialization.

## Conclusion

Throughout the second half of the 20th century, sexual attitudes have become more liberal, and since the late 1970s, there has been an egalitarian standard regarding premarital sex. These cultural changes and the inconsistent nature with which the sexual double standard is displayed favor the lack of agreement about its existence. This study contributes to the understanding of the factors that favor the inconsistency with which SDS is manifested. We conclude from our results that in order to study SDS adherence, it is necessary to consider different leves of analysis (e.g., individual and contextual). This approach will shed more light to the conditions under which the different SDS typologies occur (e.g., man-favorable SDS, woman-favorable SDS, egalitarian, and ambivalent). Likewise, contextualizing the study of SDS in the setting of relations among men and women and the motivation towards ingroup favoritism will foster a deeper understanding of the predictive role of gender, gender identity, age, dominance orientation, and sexual gender norms on SDS adherence typologies. Democratic societies favor the prevalence of egalitarian people, but SDS has not been eliminated from society, not even from democratic ones. Depending on whether the social context approves of sexual openness or censures sexist prejudices, new forms of adherence to SDS may appear and, in between, there remains an SDS that preserves the traditional gender-based social hierarchy. Understanding which conditions favor the internalization of attitudes favorable to sexual gender equality is a primary objective.

## Data availability statement

The raw data supporting the conclusions of this article will be made available by the authors, without undue reservation.

## Ethics statement

The studies involving human participants were reviewed and approved by Ethics Committee on Human Research of the University of Granada. The patients/participants provided their written informed consent to participate in this study.

## Author contributions

CG-B: conceptualization, formal analysis, methodology, project administration, resources, investigation, original draft preparation, and writing-review and editing. NM: data curation, formal analysis, methodology, software, visualization, original draft preparation, and writing-review and editing. AÁ-M: conceptualization, data curation, methodology, software, original draft preparation, review, and editing. JS: conceptualization, funding, acquisition, project administration, supervision, validation, original draft preparation, and writing-review and editing. All authors contributed to the article and approved the submitted version.

## Funding

This study was funded through a research project grant from the Ministerio de Economía y Competitividad of Spain (grant number PSI2014-25035-R) and by Bursaries FPU16/04429 for University Professor Training of AÁ-M’s (Psychological Doctoral Program B13 56 1; RD 99/2011).

## Conflict of interest

The authors declare that the research was conducted in the absence of any commercial or financial relationships that could be construed as a potential conflict of interest.

## Publisher’s note

All claims expressed in this article are solely those of the authors and do not necessarily represent those of their affiliated organizations, or those of the publisher, the editors and the reviewers. Any product that may be evaluated in this article, or claim that may be made by its manufacturer, is not guaranteed or endorsed by the publisher.
